# Notch gain of function inhibits chondrocyte differentiation via Rbpj-dependent suppression of *Sox9*

**DOI:** 10.1002/jbmr.1770

**Published:** 2013-02-15

**Authors:** Shan Chen, Jianning Tao, Yangjin Bae, Ming-Ming Jiang, Terry Bertin, Yuqing Chen, Tao Yang, Brendan Lee

**Affiliations:** 1 Department of Molecular and Human Genetics, Baylor College of MedicineHouston, TX, USA; 2 Howard Hughes Medical InstituteHouston, TX, USA

**Keywords:** CHONDRODYSPLASIA, AXIAL SKELETON, GENETIC MOUSE MODEL, NOTCH SIGNALING, *SOX9*, CHONDROCYTE

## Abstract

Notch signaling plays a critical role during development by directing the binary cell fate decision between progenitors and differentiated cells. Previous studies have shown sustained Notch activation in cartilage leads to chondrodysplasia. Genetic evidence indicates that Notch regulates limb bud mesenchymal stem cell differentiation into chondrocytes via an Rbpj-dependent Notch pathway. However, it is still unknown how Notch governs chondrogenesis in the axial skeleton where Notch serves a primary patterning function. We hypothesized that both Rbpj-dependent and Rbpj-independent Notch signaling mechanisms might be involved. Cartilage-specific Notch gain-of-function (GOF) mutant mice display chondrodysplasia accompanied by loss of *Sox9* expression in vertebrae. To evaluate the contribution of an Rbpj-dependent Notch signaling to this phenotype, we deleted *Rbpj* on the Notch GOF background. These mice showed persistent spine abnormalities characterized by “butterfly” vertebrae suggesting that removal of *Rbpj* does not fully rescue the axial skeleton deformities caused by Notch GOF. However, Sox9 protein level was restored in *Rbpj*-deficient Notch GOF mice compared with Notch GOF mutants, demonstrating that regulation of *Sox9* expression is canonical or Rbpj-dependent. To further understand the molecular basis of this regulation, we performed chromatin immunoprecipitation (ChIP) assays and detected the recruitment of the Rbpj/NICD transcription complex to Rbpj-binding sites upstream of the *Sox9* promoter. The association of the Rbpj/NICD complex with the *Sox9* promoter is associated with transcriptional repression of *Sox9* in a cellular model of chondrocyte differentiation. Hence, Notch negatively regulates chondrocyte differentiation in the axial skeleton by suppressing *Sox9* transcription, and Rbpj-independent Notch signaling mechanisms may also contribute to axial skeletogenesis.

## Introduction

The Notch signaling pathway plays a critical role in cell-fate decision, proliferation, differentiation, and maintenance of homeostasis in multiple tissues. The signaling from Notch ligands to Notch receptors is initiated by the release of the active Notch intracellular domain (NICD) through proteolytic events mediated by Presenilin1/2 within the γ-secretase complex. After the nuclear translocation, NICD forms a complex with Rbpj to activate the transcription of downstream targets including the Hes and Hey family of genes. The mammalian orthologs of the *Hes* and *Hey* genes include *Hes1*, *3*, *5*, *7* and *Hey1*, *2*, *HeyL*;^1^ they belong to the family of basic helix-loop-helix (bHLH) transcription factors and form homodimers or heterodimers with other bHLH proteins to regulate transcription.^2^

The dysregulation of Notch signaling underlies the pathogenesis of various developmental disorders and cancers. Recently, Hajdu-Cheney syndrome, a rare skeletal disorder characterized by osteoporosis, has been found to be caused by gain-of-function mutations in *NOTCH2*.^3^, ^4^ It has long been well established that loss of function (LOF) in *DLL3* or *JAGGED1*, both Notch ligands, or loss of function in *HES7*, *LFNG,* or *MESP2*, Notch downstream target genes, can lead to a spectrum of vertebral patterning defects such as those seen in Alagille syndrome or in spondylocostal dysostosis types I, II, and III.^5^ Interestingly, patients with these disorders also show symptoms attributable to alteration of cartilage and bone homeostasis, eg, short stature and low bone mass.^6^

A potential role of Notch in cartilage in vivo was first reported by Crowe and colleagues^7^ using a retrovirus-infection chick model. Overexpression of the Notch ligand *DLL1* in chick limb buds blocked differentiation from prehypertrophic to hypertrophic chondrocytes and resulted in shortened skeletal elements with decreased expression of *Col2a1* and *Col10a1*. In mice, Hilton and colleagues^8^ reported increased expression of *Ihh* and *Col10a1* with an elongated hypertrophic zone when *Presenilin1/2* were deleted in limb bud mesenchymal progenitor cells (*Prx1-cre; Presenilin1* ^*f/f*^*; Presenilin2*^*−/−*^). Similarly, an elongated hypertrophic zone resulted from deletion of *Notch1/2* receptors by *Prx1-cre*.^8^ In contrast, Notch gain of function in limb bud mesenchymal progenitor cells using a *Prx-1 cre*^9^ or later in osteochondroprogenitor cells with *Col2a1-cre*^10^ to activate expression of a Notch intracellular domain (ICD) cassette^11^ inhibited chondrocyte differentiation and hypertrophy with decreased expression of *Sox9* and *Col2a1*. Taken together, these studies illustrate two phases of Notch action: In the earlier progenitor pool, Notch signaling promotes chondrocyte proliferation. In committed chondrocytes, Notch inhibits chondrocyte differentiation and maturation. This is not surprising because we and others have shown similar context dependent and temporal actions of Notch during osteoblast commitment and differentiation.^8^, ^12^, ^13^

Although the Notch canonical pathway has been intensively explored, the Rbpj-independent or Notch noncanonical pathway is poorly understood. This mechanism has been suggested to integrate various signaling mechanisms, including cross-talk with Wnt and NF-kB.^14^ The contribution of the Notch canonical pathway to chondrogenesis was recently examined in a mouse model where *Rbpj* was deleted in the context of Notch gain-of-function using *Prx1-cre*.^15^ Removal of *Rbpj* largely reversed the Notch gain-of-function phenotype in the limbs and skull of Notch gain-of-function mutant.^15^ Moreover, the importance of the Notch canonical pathway in skeletal development was demonstrated in our recent study showing that the osteosclerotic phenotype caused by Notch1 ICD overactivation in osteoblasts is completely dependent on *Rbpj*-mediated Notch signaling.^12^ However, whether Rbpj is solely responsible for Notch function during axial chondrogenesis is still unknown. Given the patterning effects of Notch during somitogenesis, we hypothesized that alternative mechanisms may exist that distinguish Notch action during axial versus appendicular specification.

Sox9 is a master transcription factor that initiates the chondrogenic program in condensed mesenchymal cells.^16^ However, despite many studies on Sox9 function, much less is known about the transcriptional regulation of the *Sox9* gene. An examination of *Prx1-cre; Rbpj*^*f/f*^ mice suggested that *Sox9* may be regulated either in a direct or indirect manner via Notch signaling.^15^ Hence, dissecting the genetic interaction of Sox9 and Notch signaling and the mechanistic basis of Notch regulation of *Sox9* during chondrogenesis is warranted.

In this study, we addressed the specific role of Notch GOF in cartilage development in the axial skeleton after somitogenesis and examined the relative contribution of Rbpj-dependent Notch signaling in this context. Furthermore, we showed that Sox9 is a downstream transcriptional target of Notch signaling, demonstrating a mechanism by which Notch GOF impacts axial chondrogenesis.

## Materials and Methods

### Animals

Conditional knockout mice *Rbpj*^*f/f*^, *Col2a1-Cre* transgenic mice, and *Rosa*^*Notch1 ICD*^ PCR genotyping have been described previously.^13^ Animals were used in accordance with the National Institutes of Health Guide for the Care and Use of Laboratory Animals. All mice were housed in a specific pathogen-free facility and under light-, temperature-, and humidity-controlled conditions. These studies were approved by the Baylor College of Medicine Institutional Animal Care and Use Committee (IACUC).

### Skeletal preparation and histology

Whole-mount skeletal preparations were stained with Alcian blue 8GX (Sigma-Aldrich, St. Louis, MO, USA) and Alizarin red S (Sigma-Aldrich) as described previously.^17^ Littermates of control, *Rosa*^*Notch1 ICD*^*; Col2a1-cre* mutant or *Rosa*^*Notch1 ICD*^*; Col2a1-cre; Rbpj*^*f/f*^ mice were euthanized at E18.5 and whole skeletons were fixed in 10% neutral-buffered formalin overnight. Paraffin-embedded nondecalcified bones were sectioned to a 6-µm thickness and sections stained with H&E following standard protocols. Skeletal preparations were photographed with a Nikon 5 megapixel digital camera mounted atop a Nikon SMZ1500 dissection microscope. All microscope and camera settings were identical for capturing the objects compared within a group. Histology and fluorescent image files were analyzed by Axiovision software (Carl Zeiss Vision, Munchen-Hallbergmoos, Germany).

### Cryosection and immunostaining

E13.5 and E15.5 embryos of control, *Rosa*^*Notch1 ICD*^*; Col2a1-cre* mutant and *Rosa*^*Notch1 ICD*^*; Col2a1-cre; Rbpj*^*f/f*^ mice were embedded immediately in optimal cutting temperature (OCT) compound (Tissue-Tek catalog no. 4583, Torrance, CA, USA). OCT blocks were preserved in a–80°C freezer and sectioned with LEICA CM 3050S into 10-µm thickness. For immunostaining, standard procedures were followed as described earlier.^17^ Sox9 antibody (AB5535 Millipore Rabbit, Billerica, MA, USA) was diluted 1:200 and the second antibody, Goat-anti-rabbit conjugated with Alex Fluor594 was diluted 1:600. Negative control was achieved by staining the cryo-sectioned slides with secondary antibody alone.

### In vivo biotinylation ChIP (chromatin immunoprecipitation) assay

A biotin acceptor followed by a TEV protease cleavage site is incorporated at the 5’ end of Rbpj, Notch1 ICD (N1ICD), or Notch2 ICD (N2ICD). BirA, the *E. coli* biotin transferase, conjugates biotin to the biotin acceptor, which can be captured by Streptavidin beads. TEV protease is applied to separate the protein from Streptavidin beads and the ChIP assay proceeds according to the standard protocol. The cells were transfected with Piggybac-based vectors to insert three transgenes into the genome; the transgenes are rtTA (Tetracycline responsive transactivator), BirA (biotin transferase) and Rbpj, Notch2 ICD, or Notch1 ICD tagged with BTEV peptide (biotin acceptor with TEV cleavages site). A Tet-on expression system was employed to induce transcription of Rbpj or NICD with Doxycyclin (Sigma-Aldrich, D9891–25G) administration.

ATDC5 cell lines were treated with Doxycycline (Sigma-Aldrich, D9891–25G) 6 hours before collection for ChIP assay. Fixation and nuclear cell lysate extraction procedures are as described before.^18^ In brief, nuclear lysate was sonicated with BioruptorXL for 24 cycles; each cycle consisted of a 30-second sonication followed by a 30-second pause. Cell debris was cleared by centrifugation at 14,000 rpm for 5 minutes, and the cleared lysate was diluted 2.5 times with dilution buffer. Blocked T1 beads (100 µL) were added to the sonicated lysates and further rotated overnight at 4°C. Streptavidin beads (Dynabeads MyOne Streptavidin T1, Invitrogen, Carlsbad, CA, USA) were washed following standard ChIP washing conditions with four additional washes containing 1% SDS/TE at room temperature and 1% Triton X-100/TE, 0.1% Triton X-100/TE, TEV buffer at 4°C. Streptavidin beads were pulled down magnetically and digested with TEV (AcTEV Protease, catalog no. 12575–015, Invitrogen) at room temperature for 2 hours. Supernatants were collected and underwent reverse cross-linking followed by DNA precipitation with phenol-chloroform.

The *Sox9* gene coordinate and transcription start site positions were obtained through the UCSC browser. The region 6.8 kb upstream and 0.5 kb downstream of the transcription start site was uploaded to Matinspector software from the Genomatrix software suite (an online analysis tool warehouse). After selecting Rbpj as the target transcription factor, the binding sites of Rbpj were returned by the software and located back onto genomic coordinates. Primers flanking 100 bp 5’ or 3’ to the binding sites were designed for PCR and qPCR using Primer 3 software.

### RNA extraction and quantitative reverse transcription PCR analyses

RNA extractions, first-strand cDNA synthesis, and real-time PCR were carried out as previously described.^17^ Briefly, total RNA was extracted from costal cartilage of E18.5 mice (*n* = 3) with TRIzol reagent (Invitrogen). A Superscript III First Strand RT-PCR kit (Invitrogen) was used to synthesize cDNA from extracted RNA. We performed real-time quantitative PCR amplifications in a LightCycler v 1.5. β2-microglobulin was used as a reference gene controlling for cDNA quantity variation among samples subjected to real-time PCR assays. Gene expression was determined by the comparative CT method.

### Statistical analyses

Statistical significance (*p* value) was computed by using Student’s *t* test. A *p* value less than 0.05 was considered to be statistically significant. Box plots were generated by using the statistics package SPSS (IBM, Armonk, NY, USA).

## Results

### Removal of Rbpj-dependent signaling partially rescues axial skeletal abnormalities caused by Notch gain of function

A previous study demonstrated that Notch gain of function in cartilage causes a chondrodysplasia phenotype.^19^ Genetic rescue experiments showed that Rbpj-dependent Notch signaling accounts for Notch gain-of-function phenotype in the appendicular skeleton.^15^ However, it is unclear how Notch signaling regulates chondrocyte differentiation during axial skeletal development.

**Fig 1:**
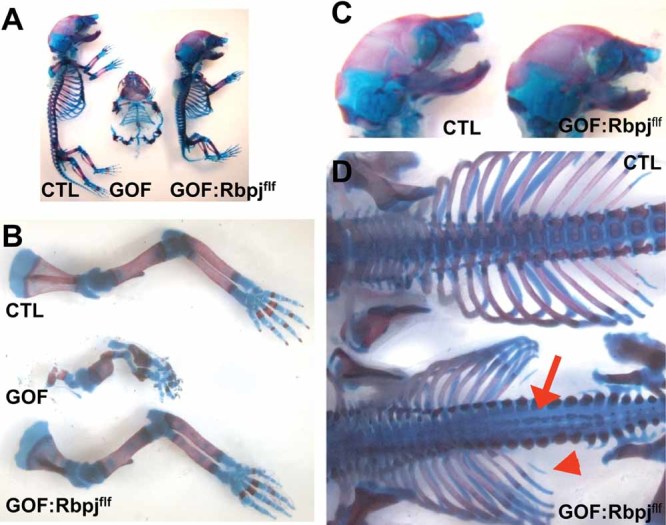
Cartilage-specific deletion of *Rbpj* in Notch GOF mice rescues appendicular but not axial skeleton structure. (*A*) Alcian blue and Alizarin red staining of skeletal preparation of control, GOF, and GOF:Rbpj^f/f^ mice. (*B*) The skeletal limb phenotypes of the GOF mice is largely rescued in the GOF:Rbpj^f/f^ mice. (*C*) Gross examination of the skull preparations suggests that GOF:Rbpj^f/f^ mice have comparable skull morphology to controls. (*D*) Examination of the spines revealed that GOF:Rbpj^f/f^ mice have an abnormal vertebral development, including a sagittal clef throughout the lumbar vertebrae (red arrow) and reduction or absence of mineralization in the smaller ribs (red arrowhead).

To understand the in vivo function of Notch signaling during axial chondrogenesis, we generated a Notch gain-of-function (GOF) model by crossing a conditionally activated Notch allele^11^ encompassing a stop cassette upstream of the activated form of the Notch1 intracellular domain (*Rosa*^*Notch1ICD*^) with a transgenic mouse line that expresses cre recombinase in cartilage under a *Col2a1* promoter (*Col2a1-cre*).^10^ The Notch GOF mice (*Rosa*^*Notch1ICD*^; *Col2a1-cre*) developed severe chondrodysplasia and exhibited perinatal lethality. These mice were characterized by craniofacial and trunk alterations, shortened limbs and tail, and prominent abdomen (data not shown), consistent with a previous report.^19^ Alcian blue and Alizarin red staining of the skeletal preparations revealed rudimentary cartilage in the limb and spine and decreased mineralization of rib cartilage (Fig. 1*A*). However, the skeletal elements formed through intramembranous ossification (such as in the clavicles and in the skull) showed mineralization comparable to control littermates. This showed that the overexpression of Notch1 ICD in cartilage impairs endochondral ossification.

Next, to determine whether the Notch GOF phenotype was solely dependent upon the *Rbpj*-mediated Notch signaling pathway, we intercrossed *Rbpj* conditional knock-out^41^ mice (*Col2a1-cre*; *Rbpj*^*f/f*^) with Notch GOF mice, so as to generate *Rbpj* deficiency on the Notch GOF background. These mice (*Rosa*^*Notch1ICD*^; *Col2a1-cre*; *Rbpj*^*f/f*^) had conditional *Rbpj* deletion as well as Notch GOF and are hereafter referred to as GOF:Rbpj^f/f^ mice. If *Rbpj*-dependent Notch signaling accounts for all Notch effects in this model, we would expect a complete rescue of the Notch GOF mice, much like we previously reported in bone.^12^

Gross examination of skeletal preparations demonstrated that the GOF:Rbpj^f/f^ mice were comparable to control littermates, although smaller (Fig. 1*A*). Indeed, the skeletal deformities in the limbs of GOF mice were rescued in the GOF:Rbpj^f/f^ mice (Fig. 1*B*). The GOF:Rbpj^f/f^ mice also had craniofacial development comparable to control littermates (Fig. 1*C*). However, examination of the GOF:Rbpj^f/f^ mice revealed persistent spinal abnormalities (Fig. 1*D*). These included a sagittal cleft spanning the cervical, thoracic, and lumbar vertebrae. Alizarin red staining showed loss of mineralized bone in the midline of the vertebral bodies. The rib cage had normal number of ribs: 13 pairs, but they appeared smaller in size. Alizarin red staining was reduced or lost in the last pair of ribs (red arrowhead) (Fig. 1*D*). In the sacrum and tail, there was also evidence of decreased mineralization (data not shown). Because no alterations in vertebral number or vertebral fusion were found, somitic patterning was undisturbed in the GOF:Rbpj^f/f^ mice, perhaps owing to the later onset of NICD expression and *Rbpj* inactivation after definitive vertebral patterning. These observations suggested a deficiency of chondrocyte differentiation and bone mineralization in the axial skeleton. Taken together, the differences between the GOF:Rbpj^f/f^ mice and control littermates suggested that *Rbpj*-dependent Notch signaling alone does not account for all of the effects of Notch gain of function during axial cartilage development.

**Fig 2:**
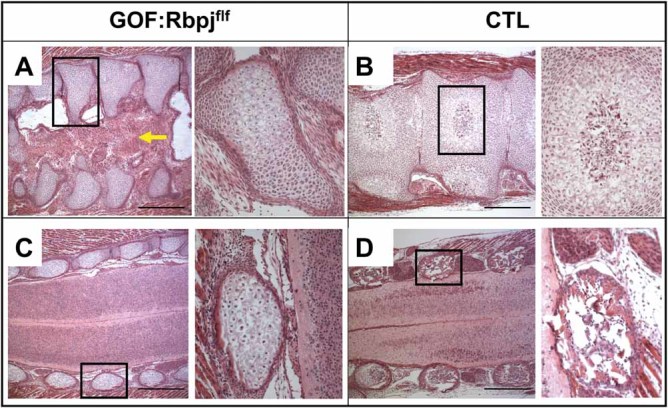
Rbpj-deficient Notch GOF mice display butterfly vertebrae. (*A*) Coronal sections of spine of GOF:Rbpj^f/f^ and (*B*) control littermate. GOF:Rbpj^f/f^ mice display “butterfly vertebrae,” fewer hypertrophic chondrocytes, and fibroblastic changes in the sagittal cleft as indicated by the yellow arrow. (*C*, *D*) Sections anterior to the vertebral body and through the lateral spinal processes of GOF:Rbpj^f/f^ and controls. Enlarged images from black boxes are shown to the right, where the vertebral bodies and the lateral spinal processes show fewer hypertrophic chondrocytes and absence of bone marrow in the GOF:Rbpj^f/f^ mice compared with control littermates.

To further identify microscopic anatomical changes in the cells and tissues of the GOF:Rbpj^f/f^ mice, histological analysis of the spine was performed. Coronal sections of the GOF:Rbpj^f/f^ mice revealed “butterfly” vertebrae consisting of two symmetrical, half vertebral bodies that were formed laterally with a wide intervening cleft (Fig. 2*A*) when compared with control littermates (Fig. 2*B*). Fibroblastic cells were found in the vertebral cleft (Fig. 2*A*). In the sacrum, there was no separation of the vertebral body, but there was a reduction of hypertrophic chondrocytes and total absence of bone marrow as no cavity formed in the centrum (data not shown).

The hemivertebrae of GOF:Rbpj^f/f^ mice showed an abundance of immature chondrocytes, reduced chondrocyte hypertrophy, and accumulation of resting/proliferating chondrocytes (Fig. 2*A*). Sections anterior to the vertebral body and through the lateral spinal processes confirmed a reduction of hypertrophic chondrocytes in the GOF:Rbpj^f/f^ mice (Fig. 2*C*) compared with control littermates (Fig. 2*D*). Together these data demonstrate that chondrocyte hypertrophy was delayed in the vertebrae of the GOF:Rbpj^f/f^ mice.

### Notch gain of function inhibits chondrogenesis via Rbpj-dependent Notch signaling

To evaluate changes in gene expression related to chondrocyte differentiation, quantitative reverse transcriptase PCR (qRT-PCR) was performed on cDNA from costal cartilage of Notch GOF and GOF:Rbpj^f/f^ mice. We elected to use rib cartilage to represent two types of chondroprogenitors: one derived from lateral plate mesoderm that gives rise to cartilage in the limb and the sternum, and the other derived from sclerotome contributing to cartilage in the spine and ribs that are attached to the sternum.^20^ Despite this heterogeneity, rib cartilage is a reliable and representative tissue for studying the mRNA expression profiles of chondrocytes without artifacts because of cell dissociation and culture.

**Fig 3:**
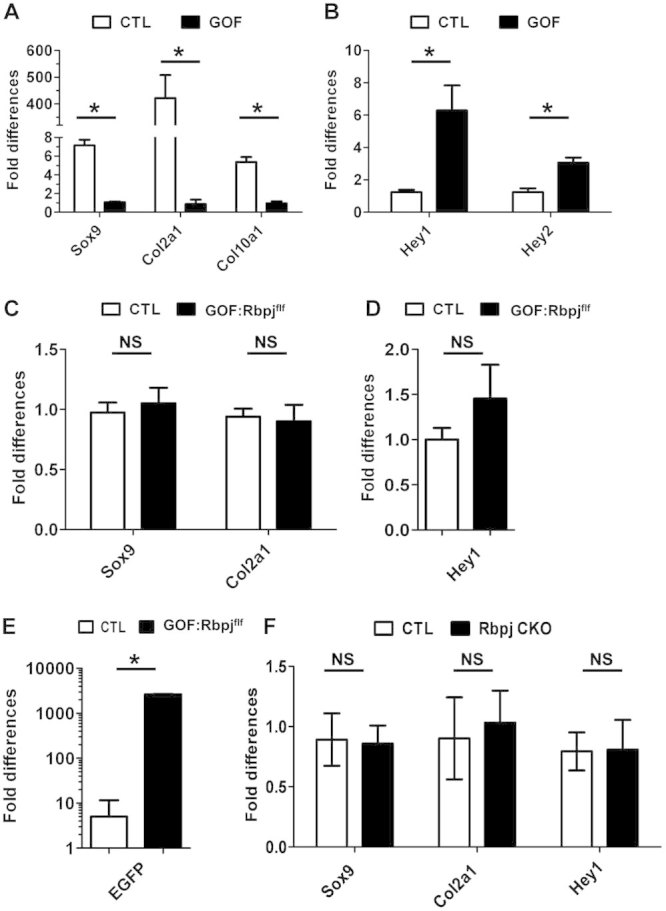
Removal of Rbpj rescues chondrocyte differentiation in Notch GOF mice. (*A*) Real-time PCR analysis of chondrocyte differentiation markers *Sox9*, *Col2a1*, and *Col10a1* in the costal cartilage of E18.5 GOF and control mice (CTL) (*n* = 3). (*B*) Real-time PCR results of Notch downstream target gene expressions (*Hey1* and *Hey2*) in the rib cartilage of E18.5 GOF and CTL mice. (*C*) Real-time PCR results of chondrocyte markers in the rib cartilage of E18.5 GOF:Rbpj^f/f^ and CTL mice. (*D*) Real-time PCR result of Notch downstream target gene *Hey1* expression in the rib cartilage of E18.5 GOF:Rbpj^f/f^ and CTL mice. (*E*) EGFP expression in the GOF:Rbpj^f/f^ and control mice. (*F*) Cartilage markers in the Rbpj CKO^f/f^ and CTL mice. *p < 0.05. NS = not significant. Error bars represent standard deviation.

The expression profiles of the *Sox9*, *Col2a1*, and *Col10a1* genes were used to assess the different stages of chondrocyte differentiation. The expression of these differentiation markers was significantly reduced in Notch GOF mice (Fig. 3*A*). Consistent with Notch gain of function, the canonical Notch targets *Hey1* and *Hey2* were upregulated in the GOF mice (Fig. 3*B*).

To evaluate the relative contribution of *Rbpj*-dependent Notch signaling to Notch GOF phenotypes at the molecular level, we assayed for cartilage differentiation markers in the GOF:Rbpj^f/f^ mice. The expression of *Sox9* and *Col2a1* was restored to levels comparable to control littermates (Fig. 3*C*), and this correlated with the robust Alcian blue staining in the ribs of GOF:Rbpj^f/f^ mice. *Sox9* and *Col2a1* expression levels were also comparable to cartilage-specific *Rbpj* knock-out mice (*Rbpj*^*f/f*^*; Col2a1-cre*, hereafter referred to as Rbpj CKO) (Fig. 3*F*). At the same time, we confirmed that *Rbpj* deletion efficiency was about 85% in the rib cartilage (Supplemental Fig. S1*A*). The level of overexpression of the *Notch1 ICD* transgene was comparable between GOF mice and GOF:Rbpj ^f/f^ mice (Supplemental Fig. S1*B*). In addition, *Sox5* expression, a transcriptional target of *Sox9*, was also significantly decreased in the GOF mice but restored in the GOF: Rbpj^f/f^ mice, consistent with the respective changes of *Sox9* in these two mutants (Supplemental Fig. S2). Corresponding to *Rbpj* deficiency in the GOF:Rbpj^f/f^ mice, elevated *Hey1* expression seen in the Notch GOF mice was restored to the wild-type levels in the GOF:Rbpj^f/f^ mutants (Fig. 3*D*), suggesting that Rbpj-dependent mechanism is involved in the Notch GOF phenotype. To exclude the possibility that the active form of NICD was not expressed or expressed at a lower level in the GOF:Rbpj^f/f^ mice, because of the introduction of additional flox sites at the *Rbpj* loci, we examined eGFP expression. In the *Rosa*^*Notch1 ICD*^ knock-in construct, the IRES-eGFP fragment is cloned downstream of NICD and when expressed it gives rise to a polycistronic mRNA carrying both eGFP and NICD; thus, the expression of eGFP is an indicator of NICD transcription. We detected robust expression of eGFP in the GOF:Rbpj^f/f^ cartilage, indicating that the restoration of chondrocyte differentiation is unlikely to be the result of decreased NICD expression independent of Rbpj deletion (Fig. 3*E*). Taken together, the molecular analyses suggested that Rbpj-dependent Notch signaling plays an important role in chondrogenesis.

### Notch gain of function inhibits *Sox9* expression in the axial skeleton

**Fig 4:**
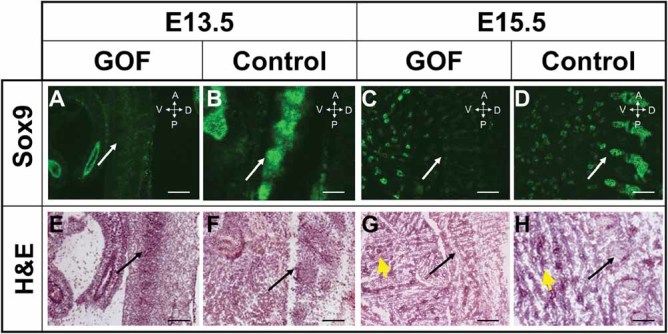
Sox9 protein expression is downregulated in vertebral bodies of the Notch GOF mice. Sox9 immunofluorescent staining on cryosections, H&E staining of adjacent sections. Sox9 staining is reduced (*A*, *C*) in the vertebral bodies (white arrows) of GOF mice compared with that of controls (*B*, *D*) at both E13.5 and E15.5. Black arrows point to the mesenchymal condensation of prospective vertebral bodies (*E*, *F*), dysplastic vertebral bodies (*G*), and normal vertebral bodies (*H*). Bronchioles in lung that express *Sox9*^39^ are denoted with yellow arrowheads (*G*, *H*). Scale bar = 250 µm. Symbols in the orientation arrows denote anterior (A), posterior (P), ventral (V), and dorsal (D).

Sox9 is an essential transcription factor required for the differentiation of condensed mesenchymal stem cells into a chondrogenic lineage. It also acts to maintain chondrocyte survival and sustain chondrocyte hypertrophy.^16^, ^42^, ^43^ Cartilage-specific *Sox9* mutants (*Sox9*^*f/f*^; *Col2a1-cre)* have severe chondrodysplasia characterized by short snouts, shortened limbs, and abnormal vertebral columns.^21^ These features are similar to our Notch GOF mice, raising the question about genetic interaction between the *Sox9* and *Notch* pathways. To assess whether Notch GOF suppresses Sox9 function, we performed immunofluorescence staining of Sox9 on frozen sections from E13.5 and E15.5 Notch GOF mutants and control littermates. Previous studies reported that *Sox9* transcript expression is abundant in condensed mesenchymal progenitor cells and is sustained in the cells progressing from resting and proliferating, and to prehypertrophic chondrocytes. It is finally downregulated in the hypertrophic chondrocytes.^22^ Here, *Sox9* protein expression was robustly detected in mesenchymal condensations (white and black arrows) in the E13.5 control mice (Fig. 4*B*, *F*), but dramatically decreased in the corresponding regions in the E13.5 GOF mice (Fig. 4*A*, *E*). Similarly, E15.5 Notch GOF mice showed no detectable Sox9 signal in presumptive vertebral condensations (Fig. 4*C*, *G*) compared with control littermates (Fig. 4*D*, *H*). As a consequence, although E15.5 control mice had normally segmented vertebral bodies, the GOF mice showed disorganized and severely reduced condensations of chondrocytes (black arrow) (Fig. 4*C–H*). These data suggest that sustained Notch activation in axial chondroprogenitors may impair chondrocyte differentiation by suppression of *Sox9*.

**Fig 5:**
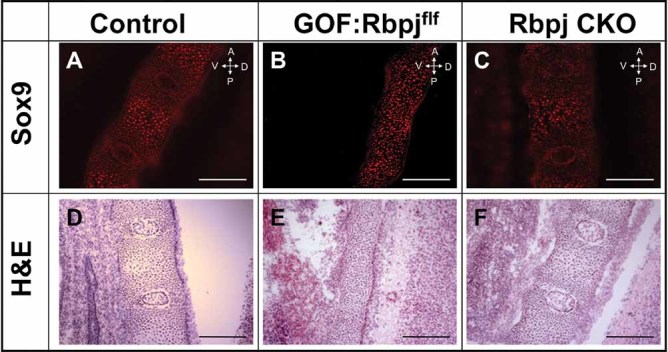
Removal of Rbpj in Notch GOF mice restores Sox9 expression in vertebral bodies. (*A–C*) Sox9 immunofluorescent staining on cryosections and H&E staining on the adjacent sections. The immunostaining reveals that the Sox9 protein level in the GOF:Rbpj^f/f^ mice (*B*) is restored to the level of control mice (*A*) or Rbpj-deficient mice (*C*). In addition, GOF:Rbpj^f/f^ mice (*E*) exhibit delayed chondrocyte hypertrophy and intervertebral disc development compared with control (*D*) or Rbpj-deficient mice (*F*). Scale bar = 250 µm.

To understand the contribution of Rbpj-dependent Notch signaling to *Sox9* expression during chondrogenesis, we assessed Sox9 protein level in the GOF:Rbpj^f/f^ mice. In the E15.5 control littermates, the vertebral bodies showed all stages of chondrocyte differentiation (Fig. 5*D*). Qualitative immunostaining showed that *Sox9* expression was concentrated in the periphery and decreased toward the center of vertebrae at the site of chondrocyte hypertrophy. Sox9 staining was also greatly reduced in the presumptive intervertebral discs (Fig. 5*A*) as the gradual reduction of Sox9 in this region is critical for patterning and development of intervertebral discs.^23^ On equivalent sections of the E15.5 GOF:Rbpj^f/f^ vertebrae, we failed to identify any hypertrophic chondrocytes, suggesting impairment in chondrocyte differentiation (Fig. 5*E*). The GOF:Rbpj^f/f^ mice also showed delayed development of the nucleus pulposus in the presumptive intervertebral discs (Fig. 5*E*). In contrast to the Notch GOF mice, we observed a qualitative restoration of Sox9 expression in the vertebral bodies of GOF:Rbpj^f/f^ mice (Fig. 5*B*), suggesting that Rbpj-dependent Notch signaling is required for *Sox9* suppression. Although Sox9-positive cells were present in the vertebral column of GOF:Rbpj^f/f^ mice at E15.5, intervertebral discs still failed to develop. We then characterized Rbpj CKO mice to determine a potential contribution of loss of Rbpj independent of Notch. If the Rbpj CKO and the GOF:Rbpj^f/f^ mice showed the same phenotype, we could conclude that pathological gain of Notch in the axial skeletal cartilage was in fact Rbpj-dependent. Otherwise, an Rbpj-independent signaling mechanism could be invoked. Unlike the GOF:Rbpj^f/f^ mice, the Rbpj CKO mice exhibited no obvious defects in chondrocyte hypertrophy and intervertebral disc development (Fig. 5*F*). Interestingly, the *Sox9* expression in vertebrae of Rbpj CKO was similar to that of the GOF:Rbpj^f/f^ and control littermates (Fig. 5*C*). Thus, even though Rbpj-mediated Notch function does not account for all of the consequences of Notch gain of function, Notch suppression of Sox9 appears to be Rbpj-dependent.

### The NICD/Rbpj transcription complex regulates *Sox9* expression by associating with the Rbpj consensus binding sites in the *Sox9* promoter

To test whether Notch suppression of Sox9 during chondrogenesis was direct, we tested whether Notch/Rbpj signaling can inhibit *Sox9* transcription in chondrocytes and whether Rbpj or Notch ICD could associate with cis-regulatory elements in the Sox9 promoter.

To assess the occupancy of the Notch/Rbpj transcription complex onto the *Sox9* promoter, we adopted and optimized an in vivo biotinylation system for ChIP (chromatin immunoprecipitation) assay. This system employs the strong affinity between biotin and Streptavidin^18^ to enhance specificity by allowing for highly stringent washing conditions (Supplemental Fig. S3). We utilized ATDC5 chondrogenic cells to generate stable cell lines in which Rbpj, N1ICD, or N2ICD could be expressed with Doxcycline administration and then subsequently biotinylated by expression of bacterial biotin transferase (BirA) in vivo.

**Fig 6:**
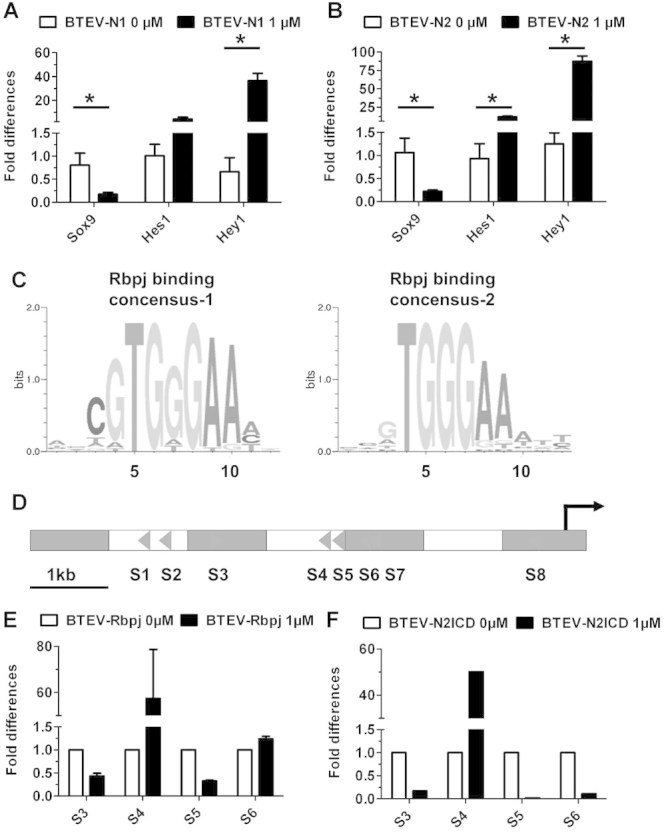
Notch downregulates Sox9 in ATDC5 cells and interacts with a specific Rbpj binding site in the Sox9 promoter. (*A, B*) Real-time PCR results show that *Hes1* and *Hey1* are upregulated, whereas *Sox9* is downregulated by either Notch1 ICD or Notch2 ICD overexpression in ATDC5 cells. (*C*) Two Rbpj consensus binding sequences shown in the form of position weight matrix (PWM). PWM is a method for representing variability or conservation of a transcription factor binding site. It is derived by tabulating the frequency with which each nucleotide is observed at each position. The “bits” on the y-axis is a measurement of the conservation of a nucleotide at a specific site. The larger the bit, the more conserved that nucleotide is at a specific site.^40^ (*D*) Predicted localization of Rbpj consensus binding sites in the Sox9 promoter. Each triangle represents one Rbpj binding site with the direction of the triangle indicating the orientation of the binding site. (*E*) Real-time PCR quantification of the occupancy of Rbpj on *Sox9* promoter after chromatin immunoprecipitation. S4, the site 3000 bp upstream of Sox9 TSS, has significant enrichment of the occupancy of Rbpj. (*F*) Real-time PCR quantification of the occupancy of N2ICD on Sox9 promoter. *p < 0.05. Error bars represent standard deviation.

As a proof-of-principle experiment, we first examined *Hes1* and *Hey1* expressions after 24-hour Dox induction in the N1ICD- and N2ICD-expressing cells (BTEV-N1 and BTEV-N2). The levels of these two genes were increased significantly compared with untreated controls (Fig. 6*A*, *B*). To test whether *Sox9* expression is altered by a gain of Notch signaling, we examined the mRNA level of *Sox9* by qRT-PCR. N1ICD and N2ICD effectively downregulated *Sox9* expression (Fig. 6*A*, *B*), supporting our in vivo findings in the Notch GOF mice (Fig. 3*A*).

Next, we tested whether *Sox9* could be regulated directly via a Notch/Rbpj transcription regulatory complex. We searched for Rbpj consensus binding sites from 6.8 kb upstream to 0.5 kb downstream of the *Sox9* transcription start site (TSS). Previous reports showed that the *Sox9* 6.8-kb upstream promoter was sufficient to drive high-level reporter activity.^24^ Utilizing the Matinspector software from the Genomatrix software suite,^25^ we identified eight Rbpj binding sites composed of two Rbpj consensus binding motifs (Fig. 6*C*) within the 6.8-kb upstream region (Fig. 6*D*). We performed Biotin-ChIP assays after a 6-hour induction by Dox in undifferentiated ATDC5 cell lines, which served as a model of chondrocyte progenitors. After ChIP-PCR analysis, we found positive bands at four sites: S3, S4, S5, and S6. We further quantified the enrichment of Rbpj or NICD occupancy at these four sites by qPCR. The mouse *Hes1* promoter (containing a canonical Rbpj binding site) was used as a positive control and a mouse *Gapdh* exonic element was used as a negative control. Because of the functional conservation of the Notch1 and Notch2 intracellular domains in vivo^26^ and the demonstrated role of Notch2 in the context of chondrogenesis,^8^ we focused our ChIP studies on Rbpj and N2ICD. According to the ChIP-qPCR result, there was significant enrichment of Rbpj and N2ICD at the S4 site 3 kb upstream of *Sox9* TSS (Fig. 6*E*, *F*). Our data suggest that the Rbpj/NICD complex can be recruited to this specific site of the *Sox9* promoter. Interestingly, the previously described transcription activation site S8 was not immunoprecipitated with either Rbpj or N2ICD in our assay,^27^ suggesting a context-dependent usage of Rbpj-binding sites in the regulation of *Sox9*.

## Discussion

Notch signaling governs cell fate determination and differentiation. However, the role of this pathway in chondrogenesis, especially in the axial skeleton, is still unclear. Our work demonstrates the critical role of Notch signaling in cartilage development and reveals the context-dependent involvement of the cofactor Rbpj in this process. We found Notch GOF inhibits chondrocyte differentiation in the committed chondrocyte progenitor by suppressing Sox9 protein expression via Rbpj-dependent Notch signaling mechanisms. However, other aspects of axial skeleton development are attributable to Rbpj-independent Notch mechanisms. Finally, we discovered that Rbpj and N2ICD are recruited to a consensus Rbpj-binding site of *Sox9* promoter to potentially mediate its repressive activity on *Sox9*.

Rbpj is a crucial mediator of Notch signaling by cooperating with NICD to regulate target gene transcription.^28^ Rbpj-independent Notch signaling mechanisms have been suggested, although the molecular basis has yet to be demonstrated in vivo.^29^ Demehri and colleagues revealed that both canonical and noncanonical Notch pathways are necessary for proper differentiation of epidermal cells.^30^ Disruption of both canonical and noncanonical Notch signaling led to a dramatic increase in pre- and immature B lymphocytes, whereas only disturbing the canonical pathway produced a modest B-lymphocyte change, suggesting that the Rbpj-independent pathway had a protective effect on restraining B-lymphocyte proliferation. Another example of noncanonical Notch signaling was brought to light by Chulan and colleagues, who demonstrated that the Notch receptor physically interacts with activated β-catenin to constrain the accumulation of β-catenin in stem or progenitor cells, independent of the ligand-mediated cleavage of the Notch receptor.^31^ Thus, both transcriptional and transcription-independent mechanisms may underlie noncanonical Notch signaling. In our study, removal of Rbpj only partially rescued the Notch GOF vertebral defects, suggesting the noncanonical Notch signaling may be involved in axial chondrocyte development. On the other hand, we cannot exclude that the remaining defects in our rescue mice could be a consequence of insufficient deletion of Rbpj, even though we demonstrated a deletion efficiency of approximately 85% of the *Rbpj* loci (Supplemental Fig. S1*A*). However, because the elevated expressions of canonical targets such as *Hey1*, *Hey2*, and *Hes1* were normalized to control levels in the GOF:Rbpj^f/f^ mice, residual Rbpj-dependent Notch signaling is unlikely to be the main cause of the residual spinal defects.

Notably, our findings in this study are supported most recently by the report by Khon and colleagues.^32^ They used a similar approach as Dong and colleagues’^15^ and ours to demonstrate that the Rbpj-independent Notch pathway regulates chondrocyte differentiation during endochondral bone development in the limb appendicular skeleton. The axial skeletal phenotype caused by the Rbpj-independent Notch pathway in our work is more severe than that of the limb, suggesting that different molecular mechanisms mediate Rbpj-independent Notch signaling in the limb versus spine. In the future, the identification of genes whose expressions do not respond to Rbpj deletion but rather respond to NICD activation would provide direct evidence for noncanonical Notch targets. Determination of the in vivo function of these target genes may give us insights on the diversified effects of Notch signaling in instructing lineage-specific and tissue-specific programs during development and diseases.

Sox9 is a master transcription factor that initiates chondrogenesis in condensing mesenchymal stem cells.^33^ However, despite the many different roles of Sox9, much less is known about how this gene is regulated. Studying the transcription regulation of *Sox9* is a difficult undertaking because human mutations that disrupt *Sox9* expression can span greater than 1 Mb up- or downstream of the *Sox9* gene.^34^ Recent studies reported that bone morphogenetic protein (BMP) and sonic hedgehog (SHH) modulate the function of Sox9 in chondrocytes in vitro, although there are no in vivo data confirming this.^35^, ^36^ Haller and colleagues demonstrated that Notch1 signaling regulates the determination of chondrocyte cell fate via *Sox9* in an in vitro assay where mouse embryonic stem cells were differentiated into chondrocytes.^27^

We propose that Sox9 is a primary Notch target based on the transcriptional downregulation in vivo and in vitro (in ATDC5 cells) as well as the recruitment of Rbpj and NICD complex onto the *Sox9* promoter. Consistent with our finding, such a direct link between *Notch* and *Sox9* has recently been implicated by several groups in their studies of the molecular mechanisms on cell fate decision and differentiation.^27^, ^37^, ^38^ In a report where Notch1 ICD specifies chondrocyte fate determination through induction of *Sox9*,^27^ a 2-kb fragment of *Sox9* promoter was analyzed to identify Rbpj consensus binding sites and then two sites located 300 bp upstream and 69 bp downstream of TSS were found. Reporter assays revealed that these two sites synergistically augment Notch-induced *Sox9* expression. Similarly, another study showed that Notch1 ICD binding to Rbpj sites 300 bp upstream of Sox9 TSS was required during bile duct differentiation.^38^

Consistent with their methodologies to identify Rbpj binding sites, the proximal Rbpj binding site 300 bp upstream of *Sox9* TSS was also identified in our study.^27^ However, our ChIP results showed that neither Rbpj nor Notch ICD was recruited to this activation site; rather they were recruited to the Rbpj consensus binding site 3000 bp upstream of TSS. This suggests that differential use of Rbpj consensus binding sites might explain the cell-context dependent and tissue-specific action of Notch signaling. This raises an interesting question of how the dimorphic regulation on *Sox9* is achieved.

Based upon our findings and those of others, we propose a model for Notch function in chondrogenesis: Overactivation of Notch in a pathological context inhibits chondrocyte differentiation in both the axial and appendicular skeleton in part by targeting Sox9 via binding of an Rbpj/NICD complex upstream in the Sox9 promoter. Moreover, both canonical and noncanonical Notch signaling pathways are important for proper axial cartilage development, whereas canonical Notch signaling predominates in the appendicular skeleton.

## Disclosures

All authors state that they have no conflicts of interest.
