# Supplementary material for: Notch gain of function inhibits chondrocyte differentiation via Rbpj-dependent suppression of Sox9
Source: J Bone Miner Res. 2013 Feb 15;28(3):649–59. doi: 10.1002/jbmr.1770 (PMC3548081; doi:10.1002/jbmr.1770)

SUPPLEMENTAL FIGURE LEGENDS

Supplemental Figure 1. Evaluation of deletion efficiency of *Rbpj* ^{flox} allele and levels of *Notch1 ICD* over-expression by *Col2a1-cre* in cartilage. A. Real-time PCR quantification shows an 85% reduction of the wild-type *Rbpj* allele present in the cartilage DNA from Rbpj CKO mutants (n=4 for each genotype). B. *Notch1 ICD* is expressed at 2.5 times higher in both GOF and GOF:Rbpj$^{f/f}$ mice compared to control mice (CTL). Primers for qPCR were designed to hybridize to homologous regions found in both human and mouse *Notch1 ICD*. Rbpj CKO: *Rbpj* $^{f/f}$ *; Col2a1-cre*. GOF: *Rosa* $^{Notch1\ ICD}$ *; Col2a1-cre*. GOF: Rbpj $^{f/f}$: *Rosa* $^{Notch1\ ICD}$ *; Col2a1-cre; Rbpj* $^{f/f}$. *: $p<0.05$.

Supplemental Figure 2. *Sox5* expression is suppressed by constitutively activated Rbpj-dependent Notch signaling in cartilage. The left panel shows *Sox5* gene expression is significantly recued in GOF mice compared to controls. However this suppression is reversed by removal of *Rbpj* as shown in the right panel. GOF: *Rosa*$^{Notch1\ ICD}$; *Col2a1-cre*. GOF: *Rbpj* $^{f/f}$*:* *Rosa* $^{Notch1\ ICD}$*; Col2a1-cre; Rbpj* $^{f/f}$. *: $p<0.05$. N.S.: not significant.

Supplemental Figure 3. Schematic diagram illustrating the principle of *in vivo* biotinylation and its application in ChIP (Chromatin immunoprecipitation) assay. The *in vivo* biotinylation over-expression system employs the exceedingly strong affinity between biotin and Streptavidin to enhance the sensitivity and specificity of the ChIP assay. A biotin acceptor peptide followed by a TEV protease cleavage site is incorporated into the 5’ end of *NICD* or *Rbpj*. BirA, the E.coli biotin transferase conjugates biotin to the biotin acceptor, which can be captured by the Streptavidin bead. The TEV protease is applied to separate the protein from Streptavidin bead

and the ChIP assay proceeds as standard protocol. This system was engineered into a murine chondrogenic cell line ATDC5, which was transfected with Piggybac integration vectors to mediate transposition of individual transgenes including *rtTA* (Tetracycline responsive trans-activator), *BirA* (biotin transferase) and *Rbpj* or *Notch2 ICD* tagged with BTEV peptide (biotin acceptor with TEV cleavages site). Because the promoter driving transcription of *Rbpj* or *Notch ICD* is inducible by Doxycyclin , we are able to fine tune the level of their expression to mimic physiological levels.

Supplemental Figure 1A. 85% of the Rbpj flox allele was deleted by Col2a1-cre

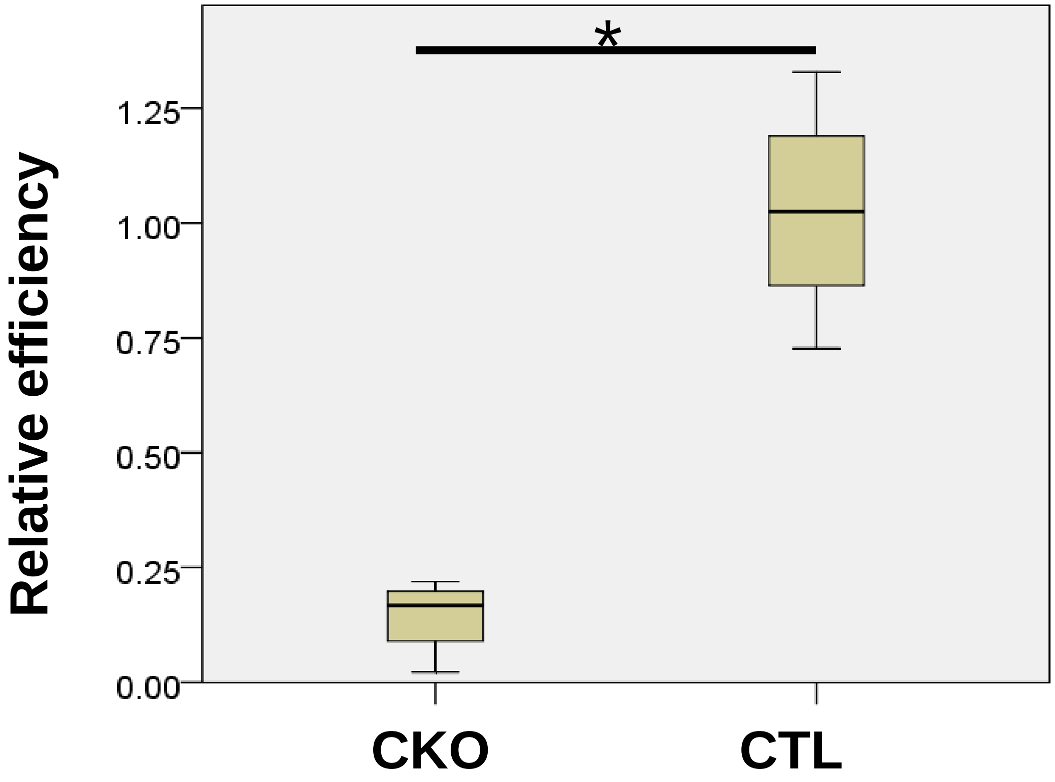

Supplemental Figure 1B. Notch 1ICD is over-expressed 2.5 times in GOF and GOF:Rbpj f/f mice compared with respective control (CTL) mice

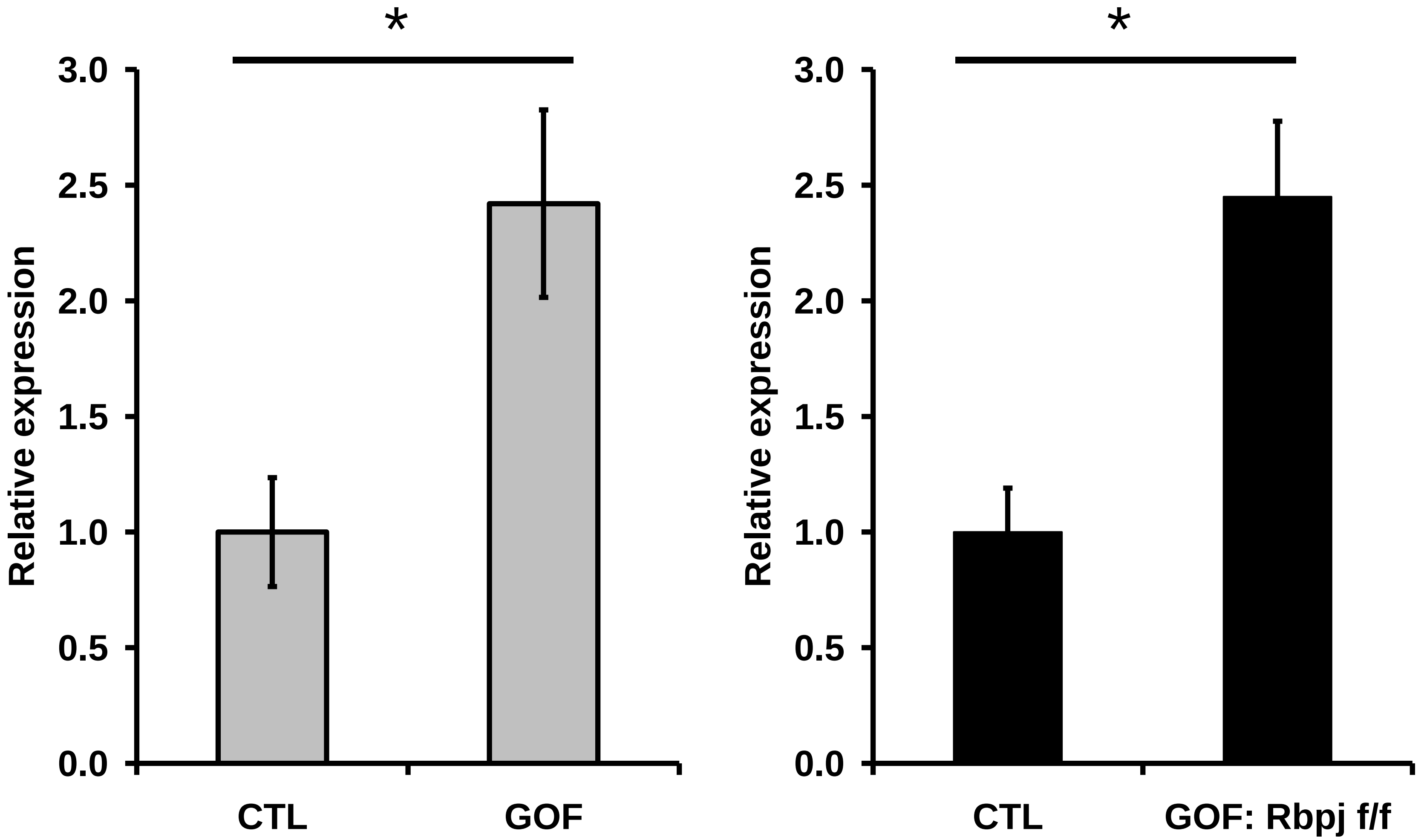

Supplemental Figure 2. Constitutively activated Rbpj-dependent Notch signaling suppresses Sox5 in cartilage.

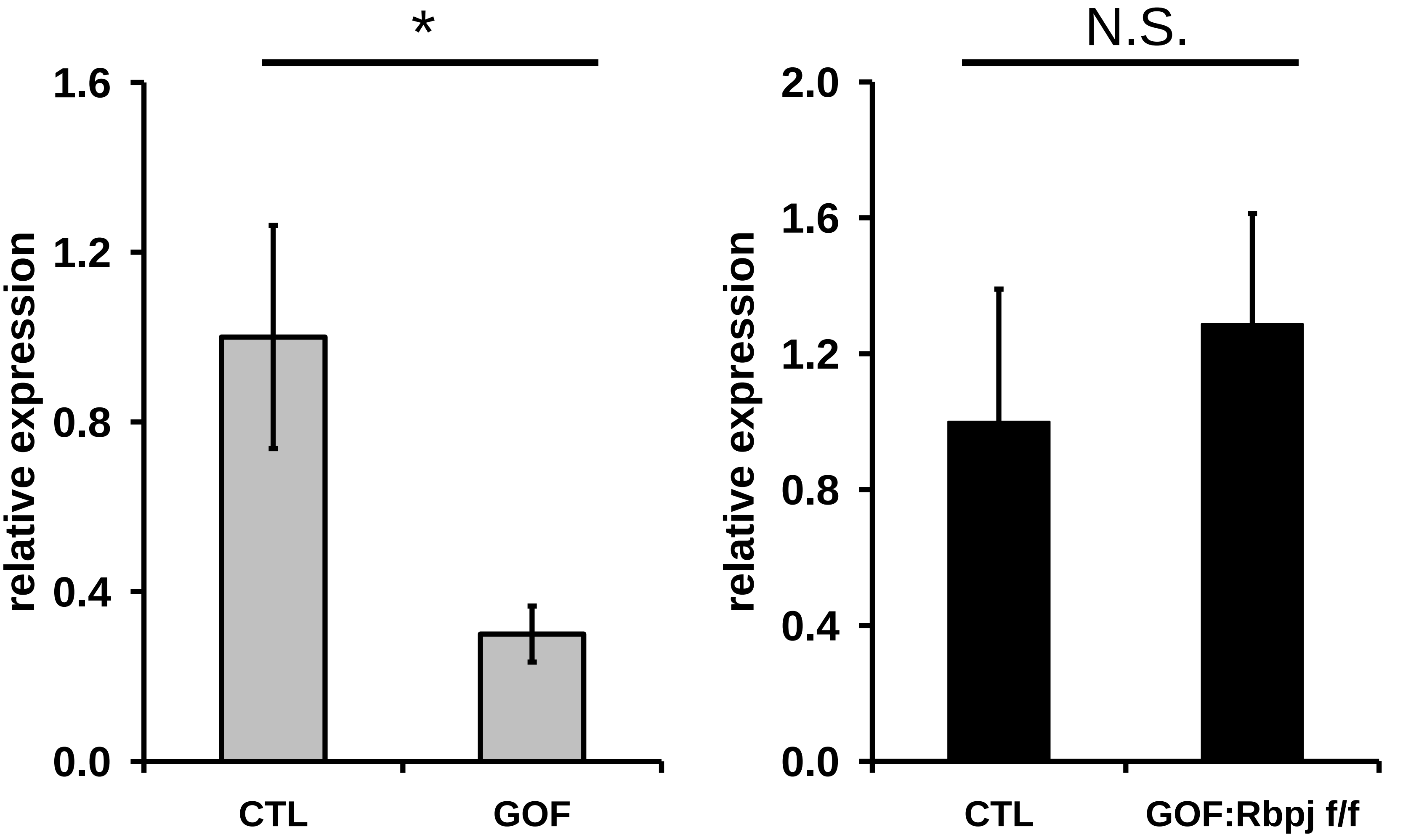

Supplemental Figure 3. Schematic of the biotinylation system

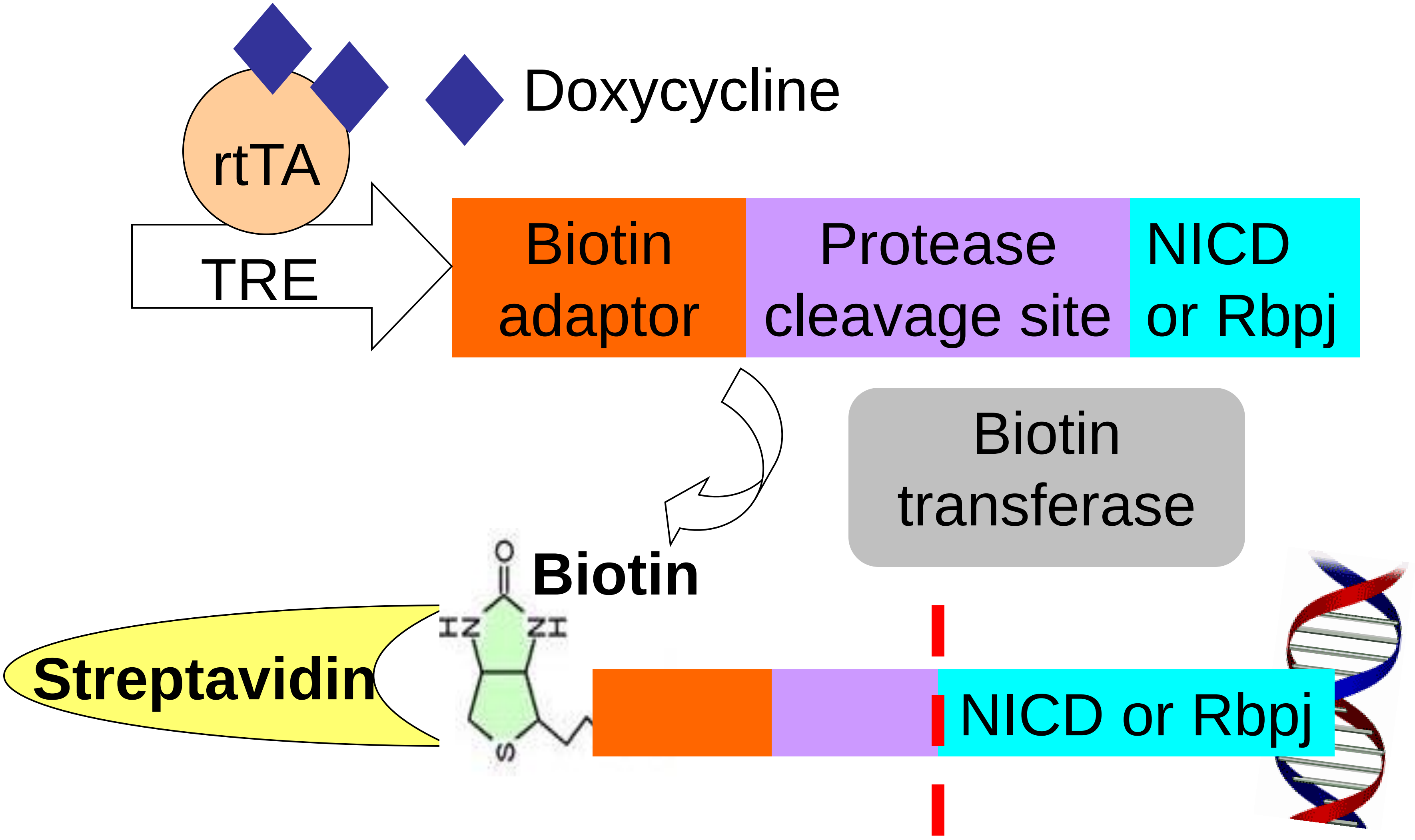

Supplement: Supplementary file 1 [file jbmr0028-0649-sd1.pdf]
